# In Vitro Model for the Evaluation of Innovative Transcatheter Debridement Device (TDD): Pericardium-Based Scaffold and Stem Cells to Reproduce Calcificated Valves

**DOI:** 10.3390/biomedicines10102352

**Published:** 2022-09-21

**Authors:** Elena Tiengo, Enrico Fermi, Ilaria Zanolla, Federica Zanotti, Martina Trentini, Enrico Pasquino, Maria Chiara Palmieri, Giorgio Soliani, Sara Leo, Elena Tremoli, Letizia Ferroni, Barbara Zavan

**Affiliations:** 1 Translational Medicine Department, University of Ferrara, 44121 Ferrara, Italy; 2 AorticLab S.R.L., Bioindustry Park, 10010 Colleretto Giacosa, Italy; 3 Medical Sciences Department, University of Ferrara, 44121 Ferrara, Italy; 4 Department of Surgery, Surgical Unit 2, Azienda Ospedaliero Universitaria of Ferrara, 44121 Ferrara, Italy; 5 Maria Cecilia Hospital, GVM Care & Research, 48033 Cotignola, Italy

**Keywords:** transcatheter debridement device, calcified valve, pericardium

## Abstract

Aortic valve stenosis has become the most common valvular disease in elderly patients. Several treatments are available such as surgical aortic valve replacement and transcatheter aortic valve implantation. To date, however, there is a need to discover alternative treatments that can delay the disease progression and, therefore, the implant of a prosthetic valve. In this regard, a decalcification procedure based on the use of ultrasonic waves could represent an innovative solution in transcatheter cardiovascular therapies. In this article, we describe an innovative transcatheter debridement device (TDD) that uses low-intensity ultrasound shock waves for calcium ablation from the native aortic valve and bioprosthetic valve. Mesenchymal stem cells were seeded onto pericardium-based scaffolds and committed into an osteogenic phenotype. After treatment with TDD, cell proliferation was analyzed, as well as lactate dehydrogenase release and cell morphology. The release of calcium and inflammation events were detected. The results confirmed that the TDD was able to induce a safe decalcification without any adverse inflammatory events.

## 1. Introduction

Aortic valve stenosis has become the most common valvular disease in elderly patients in Europe and the second most common in the United States. Epidemiological studies have established that the prevalence of aortic stenosis increases exponentially with age, with 0.2% in the population aged between 50 and 59 years, 1.3% in the 60–69 year cohort, 3.9% in the 70–79 year group, and 9.8% in the population aged between 80 and 89 years. Aortic stenosis can remain asymptomatic for a long period of time but quickly progresses after the onset of the symptoms, and the average survival can be as low as 2–3 years [1].

Stenotic aortic valves are characterized by increased leaflet stiffness and thickening due to fibrosis and calcifications and, therefore, a decreased valve opening with an increased transvalvular pressure gradient [2]. Aortic stenosis can be classified into three essential subclasses. Aortic stenosis from primary dystrophic calcification is the result of the stiffening of the leaflets and is characterized by the absence of commissural fusion to preserve the three independent leaflets. Aortic stenosis from dystrophic calcification of the bicuspid valve is characterized by a turbulent flow that is induced by the anomalous architecture of the valve that traumatizes the leaflets, which undergo sclerosis and calcification. The bicuspid aortic valve represents a predisposing factor for dystrophic calcification. Aortic stenosis from chronic rheumatic valvulitis occurs when the tricuspid architecture of the valve is preserved, but the three cusps are fused along the commissures so that the orifice is reduced to a small round or triangular central opening [3].

The severity of aortic valve dysfunction is determined by the combination of the following hemodynamic factors: peak velocity, effective orifice area, and mean transvalvular pressure gradient. A peak velocity between 2.5 and 3 m/s with a mean transvalvular pressure gradient of less than 20 mmHg and a valve opening greater than 1.5 cm^2^ is generally defined as mild aortic valve stenosis. Moderate aortic valve stenosis is defined by a peak velocity between 3 and 4 m/s, a mean gradient between 20 and 40 mmHg, and an orifice area between 1 and 1.5 cm^2^. Lastly, severe aortic valve stenosis has a peak velocity greater than 4 m/s, a mean gradient of 40 mmHg or more, and an aortic valve area less than 1 cm^2^. Patients with mild and moderate aortic stenosis tend to be asymptomatic [4].

Several treatments are at surgery disposal, such as surgical aortic valve replacement, which has been the gold standard treatment for aortic stenosis for a long time. It is an open-heart procedure performed under general anesthesia on a cardiopulmonary bypass in which an incision is made in the chest to access the heart and replace the native stenotic valve with a mechanical or bioprosthetic valve [5]. Over the last 17 years, transcatheter aortic valve implantation (TAVI) has emerged as an alternative therapeutic option for elderly patients with severe symptomatic aortic stenosis who are at high risk for open-heart surgery because of comorbid conditions and for inoperable patients. It is a technique based on a mini-invasive approach that does not require general anesthesia since the prosthetic valve is positioned inside the native valve through a catheter that, in most cases, is inserted through the femoral artery. This procedure has better mortality outcomes in one-year follow-ups and a lower incidence of atrial fibrillation and bleeding after 30 days. On the other hand, a higher incidence of moderate-to-severe regurgitation, vascular complication rates, and the need for pacemaker implantation has been found [6,7].

To date, however, there is a need to discover alternative treatments that can delay the disease progression and, therefore, the implant of a prosthetic valve. In this regard, a decalcification procedure based on the use of ultrasonic waves could represent an innovative solution in transcatheter cardiovascular therapies. There is already a well-established technique based on high-intensity focused ultrasound called lithotripsy that has proven to be highly effective for the treatment of calcific tendonitis and fragmentation of kidney stones and, recently, has been extended to the treatment of severely calcified lesions in peripheral and coronary artery diseases [8]. Similar to lithotripsy, ultrasound properly modulated in intensity, frequency, and waveform can be used to produce fractures and structural changes in aortic valve calcific deposits. For this application, much less energy density is required to limit soft tissue injury.

In this article, we describe an innovative transcatheter debridement device (TDD) that uses low-intensity ultrasound shock waves for calcium ablation from the native aortic valve and bioprosthetic valve with the aim of restoring the leaflets pliability and, therefore, regaining an adequate transvalvular flow and reducing the transvalvular pressure gradient. In addition, the device could be used to delay the implantation of the TAVI in patients aged 70–80 years for at least 2–3 years with a life expectancy longer than the TAVI average duration, thus eliminating the risk of a second operation with a high rate of death, reducing the risk of adverse events, and saving the cost of disability. The TDD decalcification capability was tested on in vitro constructs consisting of bovine pericardium-based scaffold and osteogenic differentiated mesenchymal stem cells (MSCs). Cell viability, morphology, and calcium release was analyzed, as well as inflammatory response. This study could solve the huge need for effective in vitro tests capable of testing the decalcifying properties of innovative devices.

## 2. Materials and Methods

### 2.1. Transcatheter Debridement Device (TDD)

**Figure 1:**
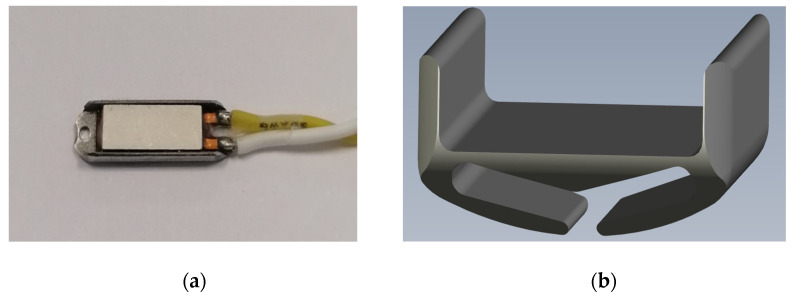
The ablation unit based on two mechanically bounded piezoceramic transducers electrically connected to a Kapton flexible printed circuit and housed in a metal support. (**a**) The piezoceramic transducer; (**b**) the metal support.

The debridement device of this experimental setting consists of an ablation unit based on a Nitinol structure on which two piezoelectric transducers are fixed. The final version of the device included an artificial, temporary valve with a Nitinol support structure intended to be positioned within the native valve to keep it open during the positioning of the transducers. The TDD was previously reported by Fermi et al. [9]. Briefly, the device comprises a pulse wave generator that provides two narrow impulsive electrical signals in the range of 90–100 V, one at 200 kHz and the other at 3150 kHz, to the ablation unit in an alternating pattern with a time interval of 6 s. The combination of these different frequencies improves the disruptive effects on calcium deposits in the aortic valve cusps while avoiding thermal injury and the breaking of the transducers. The ablation unit was based on two mechanically bounded piezoceramic transducers produced by PI Ceramics with dimensions of 2.7 mm × 8 mm × 0.7 mm (Figure 1a). Each transducer was electrically connected to a Kapton flexible printed circuit and housed in a metal support. The metal support was engineered to create a backing effect on the ultrasound emitted by the transducer to convey the waves in the direction of treatment. To obtain this effect, the thickness of the wall where the transducer was anchored was $\frac{3}{4}\lambda$ (Figure 1b). The transducer needed to be electrically isolated to work in a biological environment. It was deposited with a thickness of $\frac{\lambda }{4}$ to facilitate the passage of ultrasonic waves and limit the effects of refraction and reflection.

### 2.2. Cell Culture

Human adult MSCs (Sigma-Aldrich, St. Louis, MO, USA) were seeded on squares (1 cm^2^) of bovine pericardium membrane at a density of 5 × 10^4^ and maintained in a bone differentiation medium. The medium consisted of Dulbecco’s modified eagle medium (EuroClone), 1% penicillin/streptomycin (EuroClone, Pero, Italy), 10% fetal bovine serum (FBS, EuroClone), 10 nM dexamethasone (Sigma-Aldrich), 10 ng/mL fibroblast growth factor 2 (ProSpec), and 10 mM beta-glycerophosphate (Sigma-Aldrich) [10]. All cultures were incubated at 37 °C and 5% CO_2_ up to 21 days, and the culture medium was changed twice a week. THP-1 monocyte cell line (Resnova, Rome, Italy) was cultured in RPMI 1640 medium (Life Technologies, Carlsbad, CA, USA) supplemented with 2 mM L-Glutamine (EuroClone) and 10% FBS. Differentiation of THP-1 monocytes to macrophages was carried out by adding 100 ng/mL phorbol 12-myristate 13-acetate (PMA, Sigma-Aldrich) to the medium for 24 h. Subsequently, adhered cells were cultured without PMA for 72 h.

### 2.3. Real-Time Polymerase Chain Reaction

Total RNA was extracted with a Total RNA Purification Plus Kit (Norgen Biotek Corporation, Thorold, ON, Canada). A total of 500 ng of RNA of each sample was reverse transcribed with SensiFAST^TM^ cDNA Synthesis Kit (Bioline GmbH, Luckenwalde, Germany) following the manufacture conditions: annealing at 25 °C for 10 min, reverse transcription at 42 °C for 45 min, and inactivation at 85 °C for 5 min. A real-time polymerase chain reaction was performed with the SensiFAST^TM^ SYBR No-ROX mix (Bioline GmbH) and 400 nM primers using a Rotor-Gene 3000 (Corbett Research, Sydney, Australia). Human primers were selected by Primer 3 software. Thermal cycling conditions were as follows: denaturation at 95 °C for 2 min; followed by 40 cycles of denaturation at 95 °C for 5 s; annealing at 60 °C for 10 s; and elongation at 72 °C for 20 s. Data analysis was performed using the 2ΔΔCt method. Ct values of target genes were normalized to that of the housekeeping gene (TFRC, transferrin receptor 1). The results were reported as fold regulation of target genes in the 3D culture compared with the cells cultured on the tissue culture plate [11].

### 2.4. Cell Viability Assays

The cell viability and proliferation were detected through methyl thiazolyl-tetrazolium (MTT) assay before and after the treatment with the device [12]. The samples were incubated for 3 h at 37 °C with 1 mL of 0.5 mg/mL MTT solution prepared in phosphate-buffered saline (PBS) solution after harvesting the culture medium. Subsequently, the MTT solution was removed, and 0.5 mL of 10% dimethyl sulfoxide in isopropanol was added for 30 min at 37 °C. For each sample, absorbance values at 570 nm in duplicate on 200 μL aliquots were recorded using a multilabel plate reader (Victor 3 Perkin Elmer).

The cell membrane integrity was detected with the LDH Activity Assay Kit (Sigma-Aldrich) one and three days after the treatment. Briefly, the intracellular LDH activity was estimated after cell lysis. Each sample was incubated with a reaction mixture, and the resulting product was measured at 450 nm using the Victor 3 plate reader [12].

### 2.5. Decalcification Detection

The morphology of the cells was evaluated by scanning electron microscopy (SEM) before and after the treatment with the device. The samples were fixed with 2.5% glutaraldehyde (Sigma-Aldrich) in 0.1 M of cacodylate buffer (Sigma-Aldrich), dehydrated in ethanol, critical point dried, and then gold–palladium coated [13]. All images were obtained using a JEOL 6360LV SEM microscope (JEOL, Tokyo, Japan) at the Centro di Analisi e Servizi Per la Certificazione (CEASC, University of Padova, Padova, Italy).

### 2.6. Quantification of Secreted Factors

The Bio-Plex protein assay (Bio-Rad, Hercules, CA, USA) was used for the quantification of factors secreted by macrophages cells [14].

### 2.7. Statistical Analyses

One-way analysis of variance for data analyses was used. In addition, *t*-tests were used to ascertain significant differences (*p* < 0.05). Repeatability was calculated as the standard deviation of the difference between measurements. All testing was performed in SPSS 16.0 software (SPSS Inc., Chicago, IL, USA; license of the University of Ferrara, Ferrara, Italy.

## 3. Results

### 3.1. Shock Waves and Their Biophysical Effects

For the design of the TDD piezoelectric transducers, we follow the conclusion of recent papers [8,9], which have reported that cavitation is a fundamental phenomenon for the breakdown of calcium inside the leaflets. The variation in the radius of the bubble is inversely proportional to the frequency described above. The resonant frequency of the bubble in fluid can be expressed as [9]:

(1) $$f=\frac{1}{2\pi R\sqrt{\rho }}\sqrt{3k\left(p_{0}-p_{g}+\frac{2\sigma }{R}\right)-\frac{2\sigma }{R}-\frac{4\mu^{2}}{\rho R^{2}}}$$

where *p*_0_ is the environmental pressure, *k* the polytropic index, *R* the bubble radius, *p_g_* the gas pressure in the bubble, *μ* the density of the surrounding medium, *σ* the surface tension, and *μ* the viscosity.

The relationship can be simplified in the following way:

(2) $$R\approx \frac{3}{f}$$

The cavitation threshold is closely related to the initial bubble radius. Cavitation is induced at frequencies between kHz and MHz. The minimum frequency of a shock wave source, which induces cavitation phenomena, is given by the definition of the mechanical index or MI [15]:

(3) $$MI=\frac{P_{neg}\left(MPa\right)}{\sqrt{f}è}$$

At the peak of negative pressure, *P_neg_* corresponds to the maximum refraction of the acoustic wave. Cavitation occurs when MI > 0.7$\sqrt{f}$.

Another important element for cavitation is represented by the Bjerknes forces, which intensify the fragmentation of the bubbles due to the interaction of the bubbles with each other [16].

The following integral provides the estimate of the acoustic pressure given by the sum of the contributions of a source to *r*^1^ towards point r [17]:

(4) $$\hat{p}\left(x,y,z\right)=\frac{i\rho ck}{2\pi }\int_{S}^{}\frac{ue^{-ik\left(r-r^{1}\right)}}{\left(r-r^{1}\right)}\;ds$$

where ρ is the density of the tissue, *c* the speed of sound, *k* the wave number, and *u* is the complex surface speed.

For two excitation frequencies, the absolute value of  ρ becomes [18]:

(5) $$p_{mixed}\left(x,y,z\right)=\left|{\hat{p}}_{f1\left(x,y,z\right)}+{\hat{p}}_{f2\left(x,y,z\right)}\right|$$

where *f*_1_ and *f*_2_ represent two different frequencies of stimulation.

This demonstrates that by combining at least two different frequencies, an amplification of the effect of the ultrasound field is obtained. The combination of two frequencies accelerates bubble collapse, and cavitation bubbles become more unstable and easier to collapse under the pressure of dual-frequency ultrasound. With a dual-frequency ultrasound field, the pressure inside the bubble is higher than that obtained with a single frequency. For this reason, the dual-frequency field intensifies the cavitation effect.

### 3.2. In Vitro Model Decalcification and Integrity

In order to produce a calcific valve model in vitro, 3D constructs were produced by seeding adult human MSCs on bovine pericardium membranes. In particular, 3D cultures were maintained in an osteogenic medium for 21 days before the treatment with the TDD. Gene expression profile, morphology, and enzymatic tests were performed to test the decalcification ability of the TDD.

**Figure 2:**
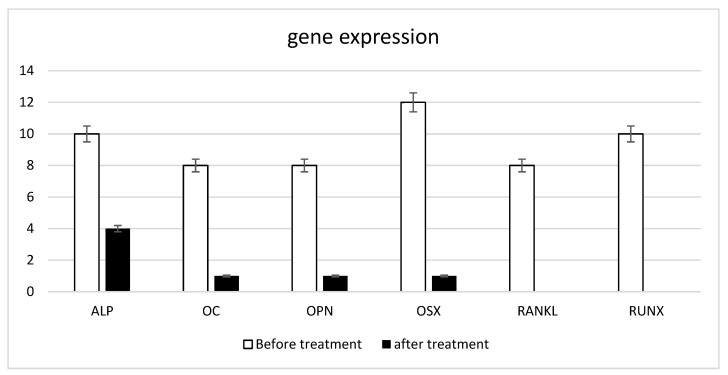
Gene expression profile of osteogenic markers: alkaline phosphatase (ALP), osteocalcin (OC), osteopontin (OPN), osterix (OSX), receptor activator of nuclear factor kappa-Β ligand (RANKL), and run-related transcription factor 2 (RUNX).

The gene expression of the typical bone markers: alkaline phosphatase (ALP), osteocalcin (OC), osteopontin (OPN), osterix (OSX), receptor activator of nuclear factor kappa-Β ligand (RANKL), and runt-related transcription factor 2 (RUNX) are reported in Figure 2 in the samples before the treatment (Figure 2, black bars). These results confirm that MSC acquired osteoblastic phenotype able to produce a well defined calcified exptracellular matrix. At 21 days after treatment (black bars, Figure 2), the gene expression was re-evaluated, and an almost complete absence of gene expression of bone differentiation markers was observed, indicating that the cells co-localizing the pericardium have returned to acquire the non-osteocytic phenotype.

**Figure 3:**
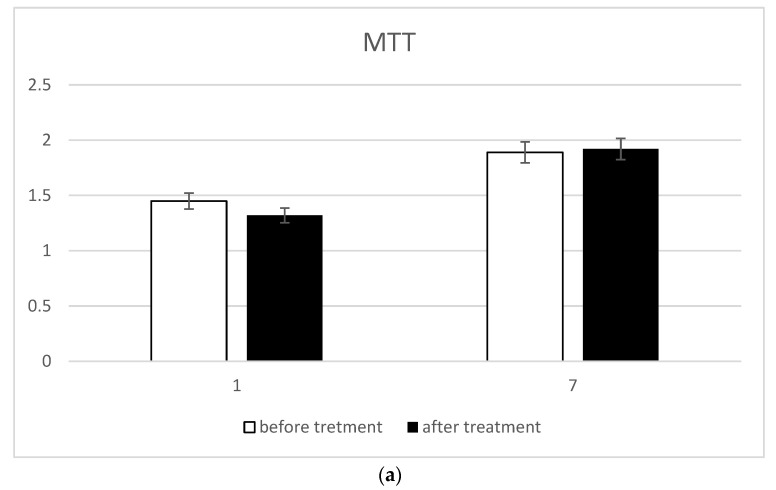

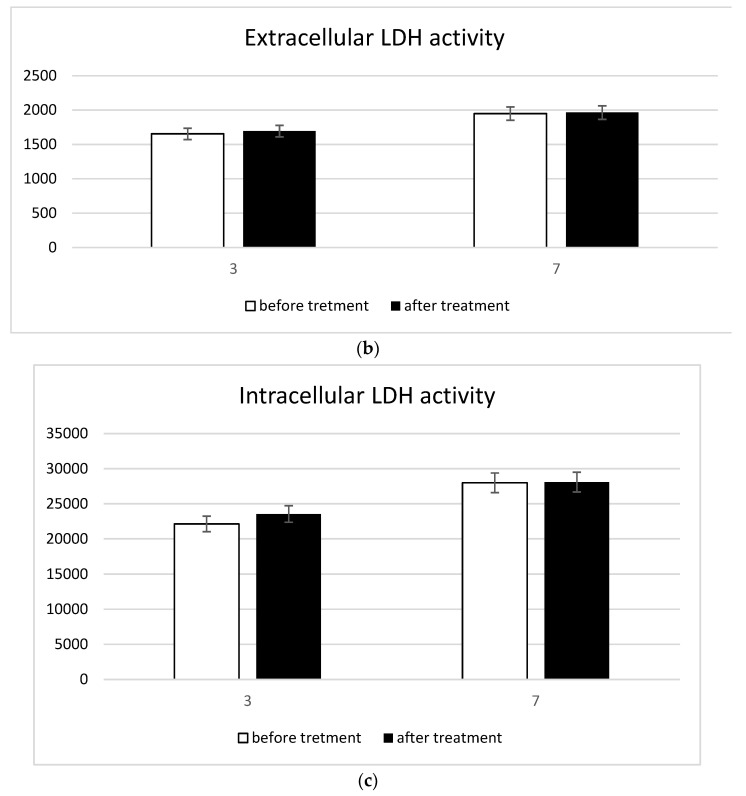
Cell integrity detection: (**a**) MTT assay; (**b**) extracellular LDH; and (**c**) intracellular LDH.

Possible treatment-induced cell damage was investigated using the MTT assays to evaluate the mitochondrial function and the detection of intracellular and extracellular LDH for the membrane damage evaluation. The mitochondrial activity detected by the MTT assay did not undergo any alterations due to the treatment (Figure 3a). Thus, cell proliferation was guaranteed even in the presence of the treatment. Furthermore, the treatment did not cause damage to cell membranes as very low extracellular LDH concentrations (Figure 3b) and very high intracellular LDH contractions (Figure 3c) were simultaneously recorded.

**Figure 4:**
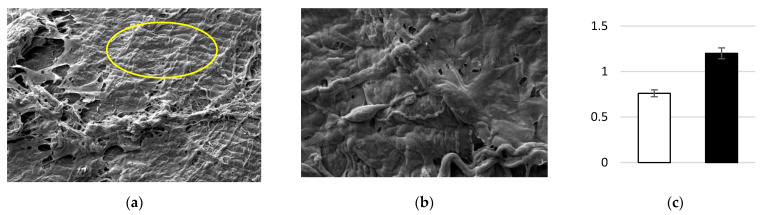
Cell decalcification detection: (**a**) SEM image before the treatment; (**b**) SEM image after the treatment; and (**c**) ARS assay before the treatment (white bar) and after the treatment (black bar).

SEM images showed the presence of a layer of calcified matrix on the osteogenic differentiated cells (yellow circle in Figure 4a). After the treatment, the calcification disappeared, and the cells on the top of the membrane were evident (Figure 4b). The treatment induced the release of the calcific component, leaving cells with a typical mesenchymal-endothelial phenotype. The release of the calcific matrix was assessed by ARS assay (Figure 4c). The amount of calcium resulting from the calcification process was markedly greater following treatment.

### 3.3. Inflammatory Response

**Figure 5:**
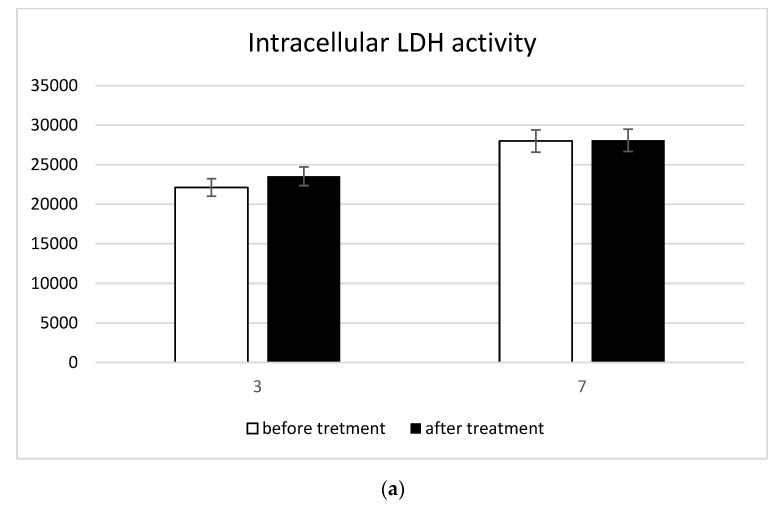

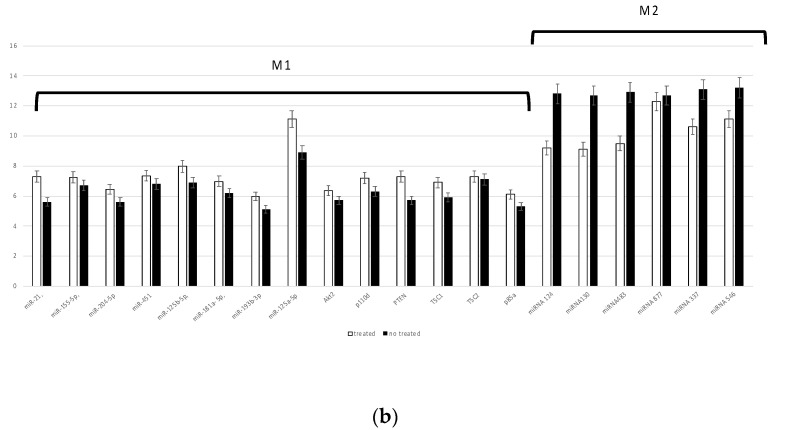
Macrophage response to the treatment. (**a**) Inflammatory cytokine release: TNF alpha, IL 1, and IL10. (**b**) Gene expression profile of M1 and M2 markers.

Undifferentiated macrophages (M0) were seeded onto the pericardium membrane and then treated with the TDD. The change into a M1 inflammatory or M2 anti-inflammatory phenotype was evaluated by cytokine release quantification and miRNA expression analysis (Figure 5). The quantification of the cytokines TNF alpha, IL1, and IL10 confirmed that the treatment induced a reduction in the inflammation process (Figure 5a). The gene expression profile of miRNA evidenced that the treatment induced an anti-inflammatory macrophage phenotype predominantly (Figure 5b).

## 4. Discussion

In the literature, the mechanism of action of ultrasound for disrupting calcific concretions is widely described [9,19]. The ultrasound field can be produced in three different ways: electro-hydraulic generator, electromagnetic generator, or piezoelectric generator [15]. In an electro-hydraulic generator, two electrodes, crossed by current, overheat the water in which they are immersed. This causes evaporation and consequently the rise in pressure that generates the shock wave. In an electromagnetic generator, a coil wrapped in a metal membrane generates a magnetic field when the current passes, which causes the membrane to expand, thus causing the formation of shock waves. The piezoelectric generator uses piezoelectric crystals, or transducers, immersed in water, which undergoes contractions and expansions of their volume, and produces very small pressure waves in the water.

For the design of the TDD, piezoelectric transducers were used to create low-intensity energy waves. The piezoelectric effect depends on the ability of a material to generate mechanical stress when subjected to a potential difference. The effect is due to the distortions suffered by the crystal lattice of the material. The transducer is like an RLC circuit; when injecting a current that has a frequency equal to the resonant frequency of the transducer, the impedance tends to zero, increasing the mechanical effects of vibration by emitting ultrasound [20]. A positive pressure pulse of short duration and a negative pressure pulse characterize the shock wave. The pressure curve that describes the shock wave is characterized by an ascending phase in which the rise time (Tr) can vary from a few nanoseconds (ns) to a few microseconds (μs) and represents the time that the pressure takes to rise from 10% to 90% of its maximum value (Pmax). The wave trend in the descending phase of the curve is instead slower and more irregular before assuming a negative value.

More in detail, the shock waves, constituted at least by two components, namely direct compression and negative tension, act on the calcific deposits with a combination of multiple effects [21]. (1) Spallation occurs when the shock wave crosses the calcification and is reflected on the back wall. The reflection of the impulse results in a mechanical tension that is more effective than the compression force (incident impulse). (2) Shear forces that result from a combination of compressive and transverse waves. This effect can be very effective on the calcific deposits since the stratified and fragile conformation of calcium concretions have a low resistance to transverse shear forces. (3) Reflection of refracted waves that are generated from the reflection of the pressure waves during the crossing of the concretion. (4) Cavitation of the bubbles consists of the nucleation of bubbles near the calcific deposits in the blood and subsequent dynamics involving growth, oscillation, and collapse with the formation of microjets. (5) Fatigue that can cause the breakage of calcific deposits when subjected to mechanical stimuli. This usually occurs where there are imperfections in which the effects of shock waves are concentrated. (6) Superfocusing is created by the geometry of the calcification as a combination of reflections and refractions of the waves that are focused within it.

As already reported by Fermi et al. [9], cavitation is a fundamental phenomenon for the breakdown of calcium that forms inside the leaflets. In fact, the TDD is based on histotripsy, an experimental cavitation-based therapy in which ultrasound “breaks” the fluid they pass through, forming bubbles of dissolved gas. The gas bubbles, subjected to the ultrasonic field, undergo compression and decompression forces, transforming the bubble into an oscillating system that can explode. The explosion of the bubble causes mechanical erosion due to the concentrated release of energy. The bubble oscillates elastically according to the gas inside it and according to the liquid surrounding it: this means the bubble has its own oscillation frequency. The cavitation threshold is closely related to the initial bubble radius. When the frequency of the ultrasound field comes close to the proper bubble frequency, resonant phenomena occur: the bubble dilates during the negative phase of the pressure wave and collapses very quickly and violently upon the arrival of the positive pressure.

The combination of multiple frequencies affects the formation of bubbles leading to the formation of a larger number of bubbles with different rays: the high frequency generates small bubbles locally, while the low-frequency stimulation generates large bubbles, and the combination of the two frequencies increases the generation rate of cavitation bubbles. The lower frequency must be approximately thirty times lower than the high frequency. This effect forms a number of bubbles that are up to five times larger compared to the use of a single frequency. Furthermore, the non-linear effects, together with the combination of the two frequencies, reduce the threshold to generate cavitation effects. Another important element for cavitation is represented by the Bjerknes forces, which intensify the fragmentation of the bubbles due to the interaction between the bubbles themselves [16]. The combination of the two frequencies accelerates bubble collapse: cavitation bubbles become more unstable and easier to collapse under the pressure of dual-frequency ultrasound. With a dual-frequency ultrasound field, the pressure inside the bubble is higher than that obtained with a single frequency. For this reason, the dual-frequency field intensifies the cavitation effect. To prevent procedure-related strokes and avoid the passage of blood clots and thrombi generated during the procedure, an anti-embolic device can be used in combination with the transcatheter debridement device (TDD). An anti-embolic filter such as Flower™ can be deployed and positioned in the ascending aorta, covering all three main branches of the aortic arch (brachiocephalic trunk, left common carotid artery, and left subclavian artery) and the systemic circulation. The filter is able to capture and remove debris during the TDD treatment, protecting patients from cerebral embolic injuries and embolism of peripheral organs.

In light of such considerations, the TDD was produced to induce an in vivo decalcification of valves. Prior to in vivo testing the device, here, we reported an in vitro system simulating calcified valves to analyze the ability of the device to induce calcification without affecting cell viability and inflammation. The system was composed of MSCs with osteogenic phenotype seeded onto bovine pericardium membrane. The ability of the MSCs to differentiate into different commitments has already been widely documented elsewhere [22,23,24]. They can differentiate toward the osteogenic phenotype in the presence of osteogenic differentiative medium [25,26,27] and several biomaterials [28,29,30,31,32,33]. Moreover, MSCs are considered a useful tool for tissue regeneration as they can release growth factors and vesicles [34]. Meanwhile, bovine pericardium membrane is widely used for tissue scaffolding [35,36] and the fabrication of artificial heart valves [37]. The current results demonstrated that the MSCs were rightly osteogenic committed and, after the treatment, cell viability was not affected. No cell membrane damage was detected after the treatment. Indeed, the high level of lactate dehydrogenase enzyme inside the cells, together with the low level on the outside, was a clear sign of an absence of damage to the plasma membranes. By contrast, a significant release of calcium from cells was observed, confirming the decalcification process. Lastly, macrophage cultures were performed to assess the inflammatory response due to the decalcification treatment. The extent of differentiation into M1 or M2 macrophages showed no onset of inflammation after the treatment.

Talking about the possibility to perform in vivo tests, we have to take into account that in vivo tests on animal models have already been performed to evaluate the navigability and usability of the transcatheter version of the device. This TDD version comprises a delivery module based on multi-lumen catheters (to carry electric signals and electrical power to the ablation units that emit ultrasound field) and an artificial, temporary valve to provide the valve function during the fragmentation treatment of the calcium deposited on the leaflets. In particular, as reported in “Lithotripsy of Calcified Aortic Valve Leaflets by a Novel Ultrasound Transcatheter-Based Device”, (1) an experimental treatment was carried out in two adult pigs in a fully equipped operating room with a portable angiographic C-arm. Under general anesthesia, the animals were fully heparinized, and the femoral artery was exposed to perform the treatment with TDD using a minimally invasive procedure. The animals were kept anesthetized for the whole duration of the treatments and were immediately euthanized after the procedure to recover the heart and dissect the treated valves. The mean arterial pressure was stable during the entire procedure (mean arterial pressure of 80 mmHg with a heart rate of ~130 beats per min). No moderate to severe aortic regurgitation was ever detected by echocardiographic monitoring during and after the TDD treatment. No pacing or rapid pacing was needed for the treatment. Efficacy tests were also performed on ex vivo models, measuring the variation of the transvalvular pressure gradient before and after treatment. The heart affected by aortic valve stenosis, explanted from a cadaver at the École de Chirurgie in Lyon, was connected to the simulation device developed by the Politecnico di Milano. (2) The device was capable of simulating the pumping function of the heart through the external pressurization of the ventricle, either in pulsatile or steady flow conditions, while measuring the transvalvular pressure drop and the flow. Pressure and flow measurements were carried out pre- and post-treatment with TDD. All the tests showed reductions in the transvalvular pressure gradient and an increase in the effective orifice area between 10% and 26% [38,39].

## 5. Conclusions

In light of such considerations, we can conclude that the TDD was able to induce a complete decalcification of the valve without affecting cell viability. Moreover, the treatment did not induce any inflammatory events able to drive a worse prognosis.

## Data Availability

The data that support the findings of this study are available from the corresponding author upon reasonable request.
